# In-bag abdominal manual morcellation versus contained power morcellation in laparoscopic myomectomy: a comparison of surgical outcomes and costs

**DOI:** 10.1186/s12893-023-02007-5

**Published:** 2023-04-28

**Authors:** Cenk Mustafa Güven, Dilek Uysal

**Affiliations:** 1 Department of Obstetrics and Gynecology, Izmir Private Can Hospital, İzmir, Turkey; 2 Department of Obstetrics and Gynecology, Atatürk Training and Research Hospital, University of Katip Çelebi, İzmir, Turkey

**Keywords:** Gynecology, Myoma, Glove in-bag morcellation, Laparoscopic myomectomy, Manual morcellation

## Abstract

**Background:**

To compare the surgical outcomes and costs of in-bag abdominal manual morcellation (AMM) and contained power morcellation (PM) in laparoscopic myomectomy.

**Methods:**

A total of 61 patients were divided into two groups based on their myomectomy specimen extraction methods: AMM group (*n* = 33) and electromechanical contained PM group (*n* = 28). The surgical outcomes and cost were compared between groups. During AMM, a glove bag (in 27 patients) and an endo bag were used (in 6 patients) according to the myoma size.

**Results:**

Morcellation time (18 ± 9.2 min vs. 37.4 ± 14.1 min) and total operation time (100 ± 24.3 min vs. 127 ± 33.1 min) were significantly lower in the AMM group compared to those in the PM group. Other surgical outcomes, which were similar between groups, included delta hemoglobin, length of hospital stay and VAS score at 12 and 24 h postoperatively. There were no per- or postoperative complications in both group with no conversion to laparotomy. One patient was transfused with two units of erythrocyte suspension postoperatively in the PM group. Sarcoma was not diagnosed in any of the cases in both group.

**Conclusion:**

The in-bag AMM or contained PM for specimen extraction resulted in similar outcomes in terms of delta hemoglobin, postoperative pain intensity (VAS score at 12 and 24 h postoperatively), the need for additional analgesia, and the length of hospital stay; however, total operation time and morcellation time were significantly shorter in the AMM group, indicating a prominent advantage. Significant cost-effectiveness is also a critical advantage of in-bag AMM compared to containing PM.

## Background

Surgical approaches are accepted as definitive treatments for uterine myomas, with myomectomy mandatory for women who wish to have children. Compared with a laparotomy, laparoscopic myomectomy is superior concerning surgical outcomes such as postoperative pain, intraoperative blood loss, length of hospitalization, and general morbidity [1, 2]. However, long operative time, high cost, and the inability to remove specimens from the laparoscope are limitation factors of laparoscopic surgery. To overcome the specimen extraction challenge, the morcellation approach was developed during which larger specimens are cut into pieces to remove them from the abdominal cavity [3].

Although morcellation can be performed manually, intraabdominal or intracorporeal PM, also known as electromechanical morcellation, has been the primary method of morcellation during laparoscopic myomectomy [4]. A power morcellator is a device that transforms electrical energy into mechanical action in the form of fast-rotating cylindrical knife movements which cut large masses of tissue into smaller pieces [4]. However, this method has introduced the risk of dissemination of the removed tissue, which could result in benign myoma seeding or spillage of malignant material into the abdominal cavity [5]. In 2014, the US Food and Drug Administration released a warning statement about discouraging the use of PM [6]. Consequently, power morcellation ceased at many institutions, and myomectomy via laparotomy increased [7]. In the forthcoming years, for continuation of laparoscopic surgery, contained PM has emerged as a means of preventing tissue spillage during extraction, with the literature supporting the claim of a low spillage risk for contained PM. It has also some restrictions, such as a longer operative time (20–30 min) compared to uncontained PM [8–11]. Both in-bag abdominal and vaginal route manual morcellation (MM) techniques have been described for laparoscopic surgery [4]. During AMM, the specimen is cut into small pieces with a knife or scissors via small abdominal wall incisions [12].

This study aimed to compare the surgical outcomes and costs of in-bag abdominal manual morcellation and contained power morcellation in laparoscopic myomectomy.

## Methods

### Study design and study group

We performed a multicenter retrospective study at the Izmir Private Can Hospital, Izmir, Turkey and the Atatürk Training, and Research Hospital, Katip Celebi University, Izmir, Turkey. Ethical approval was obtained from the local ethics committee of the Atatürk Training and Research Hospital, Katip Çelebi University, Izmir, Turkey (24.03.2022-IRB#0140), and informed consent was obtained. All protocols were conducted under the principles of the Declaration of Helsinki.

The initial cohort assessed for eligibility comprised 76 women who had undergone laparoscopic myomectomy for myomectomy indications by an experienced surgeon (CMG) from 2017 to 2022. After the first analysis of data, 61 patients were finally enrolled in this study.

Inclusion criteria were determined as follows; age between 18 and 40 years, body mass index (BMI) 18–40 kg /m^2^, and the presence of a symptomatic (heavy menstrual bleeding, pelvic pain, and infertility) single myoma measuring at least 5 cm or more.

Women who had undergone multiple myomectomies (15 patients) were excluded to standardize the total operation time. The patients were divided into two groups according to their myomectomy specimen extraction methods. All of the patients were informed about the cost of different methods of morcellation before the surgery. According to the surgeons’ and patients’ preferences, tissue extraction was performed in 33 patients were in the AMM group and 28 in the electromechanical contained PM group.

### Data collection

Preoperative assessments (recording of demographic characteristics (age, body mass index, previous pelvic surgery, type of delivery, and gravida-parity), detailed bimanual pelvic examination, a transvaginal ultrasound, pelvic magnetic resonance imaging, Ca-125 values, cervicovaginal smear, and endometrial sampling as necessary) were reported. Pelvic surgery history was considered positive in patients with a previous cesarean section, adnexal surgery, or any surgical procedure in the pelvis.

The following data were also obtained and recorded from electronic medical records: the greatest dimension of the myomas in centimeters, the localization of the myomas, intra- and postoperative complications, total operation time, morcellation time, length of hospital stay, myoma weight, postoperative pain assessment by the visual analog scale (VAS), the mean cost of morcellation and preoperative and postoperative hemoglobin levels.

The difference between preoperative and postoperative hemoglobin levels was identified as delta hemoglobin. The total operation time was defined as the time between the first incision and the extraction of the last trocar. The morcellation time was calculated as the time interval from the securing of the hemostasis of the myoma bed to the container bag being removed for each group, through the use of unedited video recordings of all cases. The length of hospital stay was defined as the number of hours of hospitalization after surgery. The postoperative pain was evaluated by VAS (1: little pain and 10: intense pain) at 12 and 24 h.

The mean cost of morcellation was defined as the cost related to the extraction method for each patient (the costs of a power morcellator, specimen retrieval bags, Endo bags, and gloves), and was calculated by dividing the total cost by patient numbers in each group according to hospital billing department records.

### Surgical approach

All patients fasted for at least eight hours before surgery; no other bowel preparation methods were employed. All laparoscopic myomectomy procedures were performed under general anesthesia while the patients were in the dorsal lithotomy position. A ClearView® uterine manipulator was placed vaginally for uterine manipulation. A Foley catheter was placed for drainage and was kept in place until the postoperative 24 h. A single shot of the first-generation 1 g of cephalosporin (2 g if the patient weighed 80 kg or more) was administered prophylactically at the time of the first incision. All patients were given 400 µcg of misoprostol rectally 30 min before the surgery [13].

**Fig. 1:**
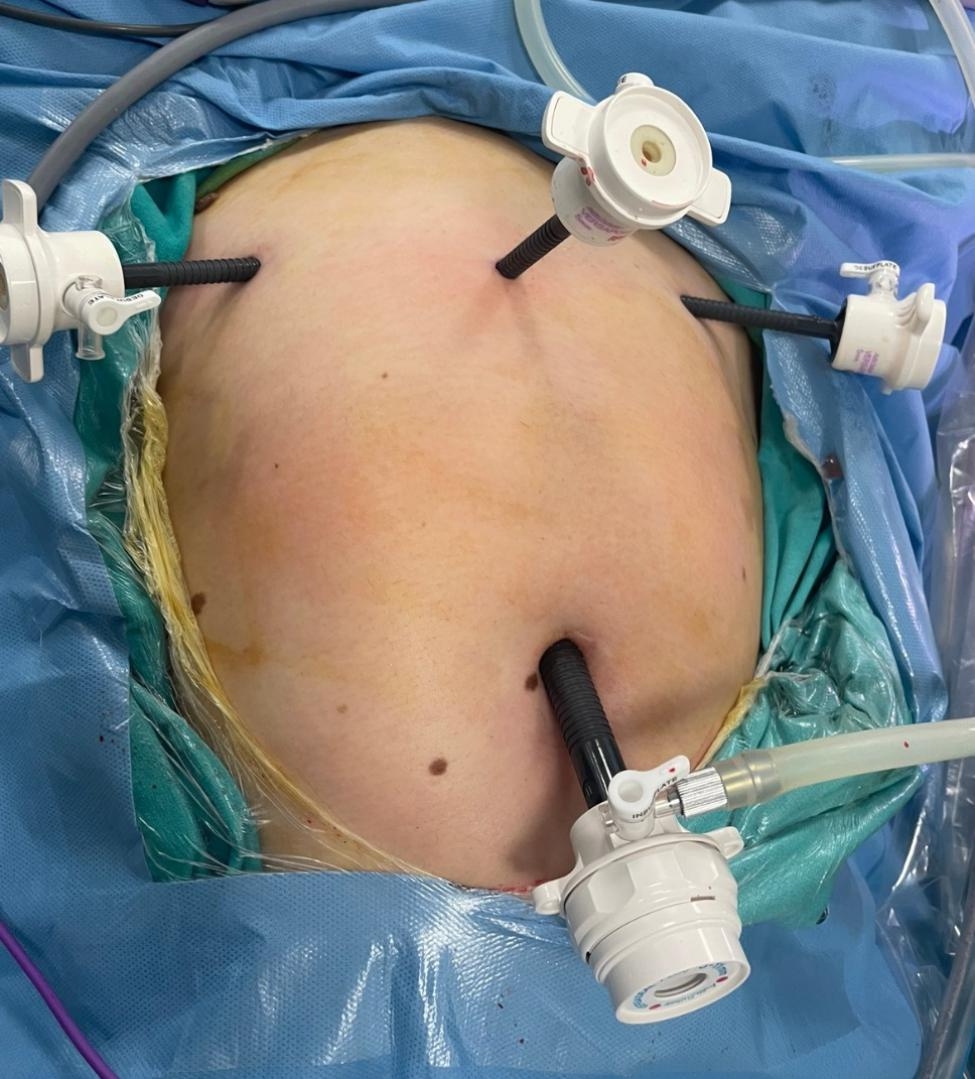
Standard port positions during operations

The standard laparoscopic myomectomy operation was conducted as follows: pneumoperitoneum was achieved from the umbilicus with a Veress needle. After enlarging the umbilical incision, a 10-mm scope was placed in the abdomen, followed by its placement in a 20^o^ Trendelenburg position. Following a routine abdominopelvic inspection, 5-mm trocar sheet were placed in both lower quadrants, approximately 3–4 cm medial to the crista iliaca anterior superior on both sides. Another 5-mm sheat was placed on the midline 6–8 cm above the symphysis pubis (Fig. 1). Following identification of the myoma, the serosa and the myoma bed were infiltrated with an isotonic saline solution. The overlying uterine serosa on the most prominent part of the myoma was coagulated longitudinally by bipolar cautery. A longitudinal incision was made on the coagulated line over the myoma by a monopolar hook. After reaching the cleavage plane, the myoma was dissected gently from its bed using both a sharp and blunt dissection. After the enucleation, the myoma bed was closed using 0 polyglactin 910 (Vicryl®) with the separated extra-corporal knotting technique in a multilayered fashion.

**Fig. 2:**
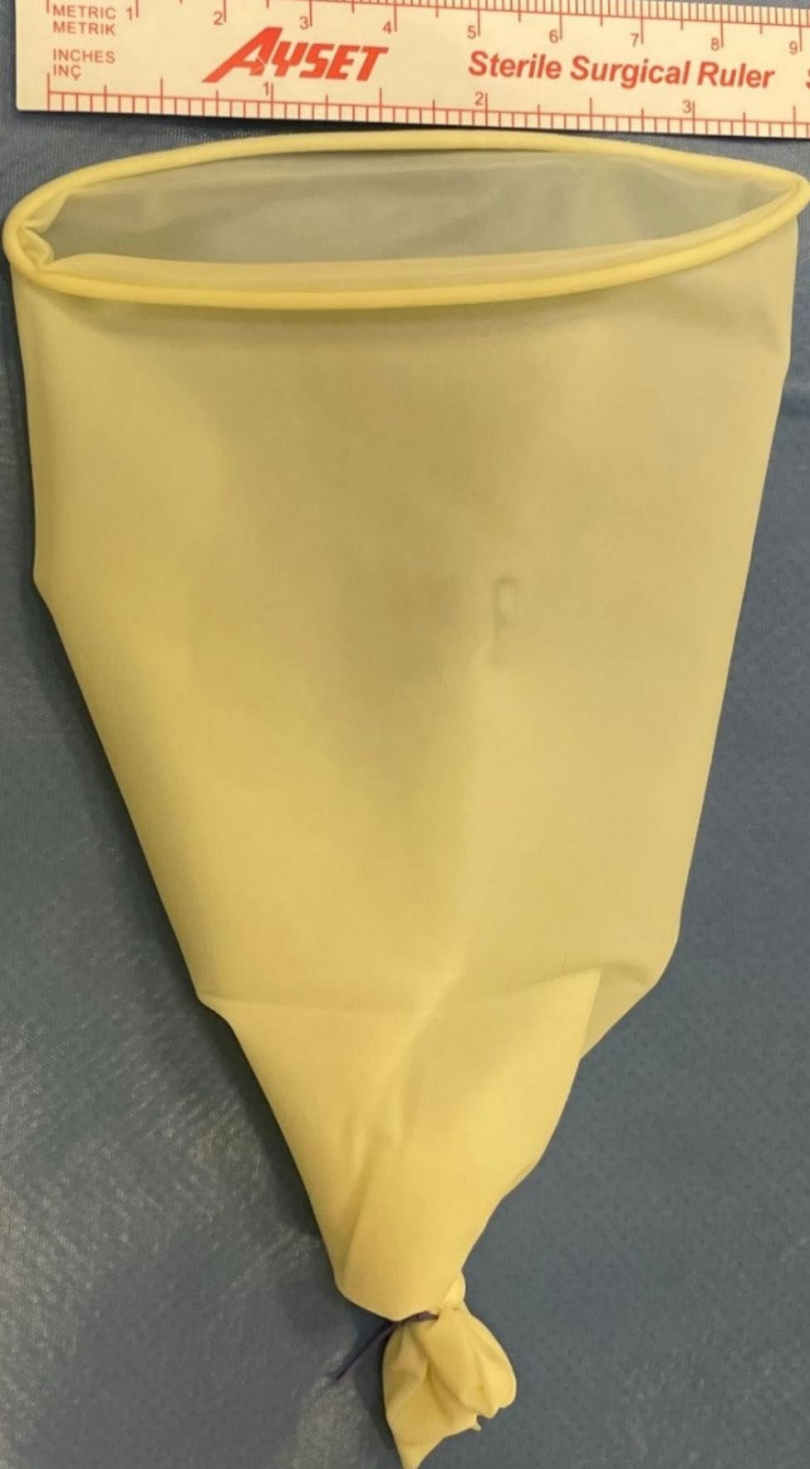
Preparation of the glove bag

**Fig. 3:**
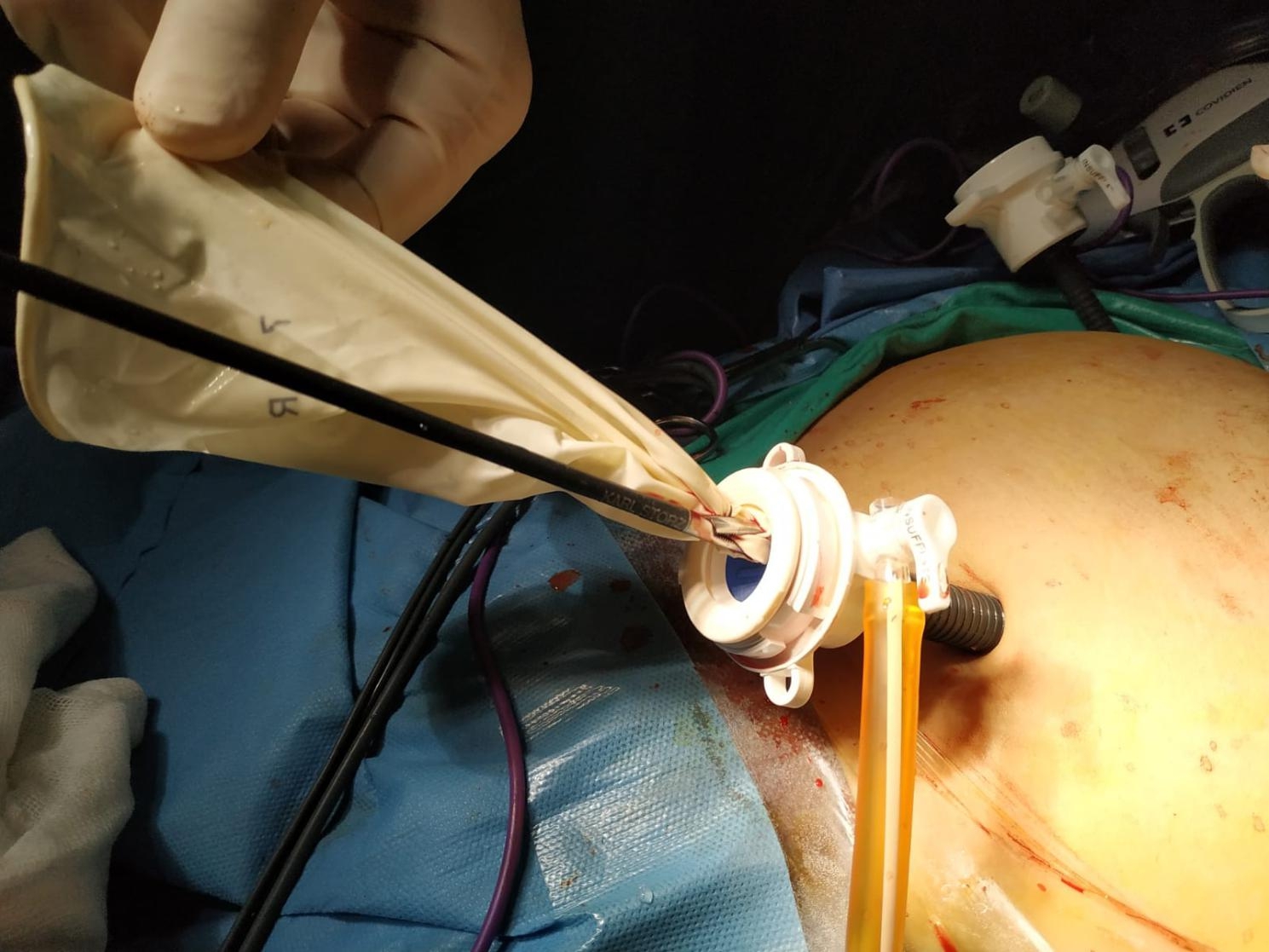
The glove bag introduced via an umbilical trocar

**Fig. 4:**
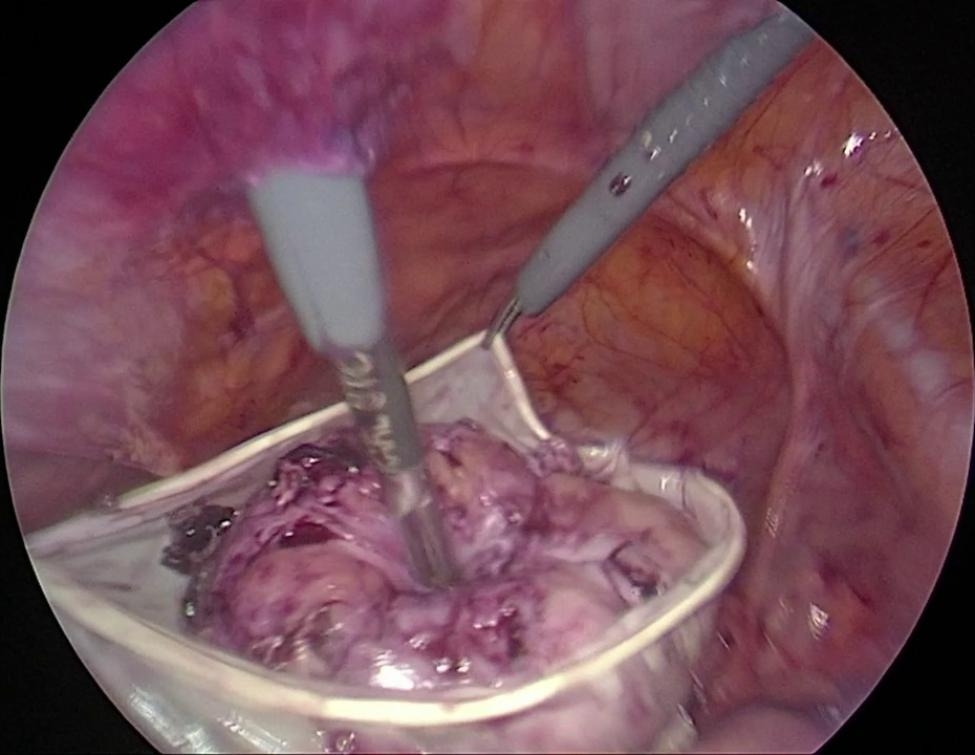
A 9 cm myoma positioned in a glove bag

After the myoma bed was sutured, a hand-made retrieval bag tailored from 8.5-sized latex-free surgical gloves (Fig. 2) was inserted into the abdominal cavity via an umbilical 10-mm sheet in women undergoing AMM (Fig. 3). The myoma was placed into the glove bag (Fig. 4). Then the left-sided 5-mm sheet was pulled out and the incision enlarged to 20 mm. A laparatomy pens was inserted into the abdominal cavity via the same incision. The jaws of the pens were widened as much as possible to enlarge the tendinous abdominal fascia. After grasping the edges of the glove bag, it was pulled out from the abdominal cavity. But in 6 patients whose myoma size was 10 cm or bigger, a commercial disposable 15 mm Endo bag (EndoCatch II Covidien®) was used instead of a glove bag. In these patients, a 12-mm trocar sheet was inserted after enlarging the left trocar incision. The myoma was put into the Endo bag and exteriorized normally. The extra costs of commercial Endo bags were added to the final cost analysis of the AMM group.

**Fig. 5:**
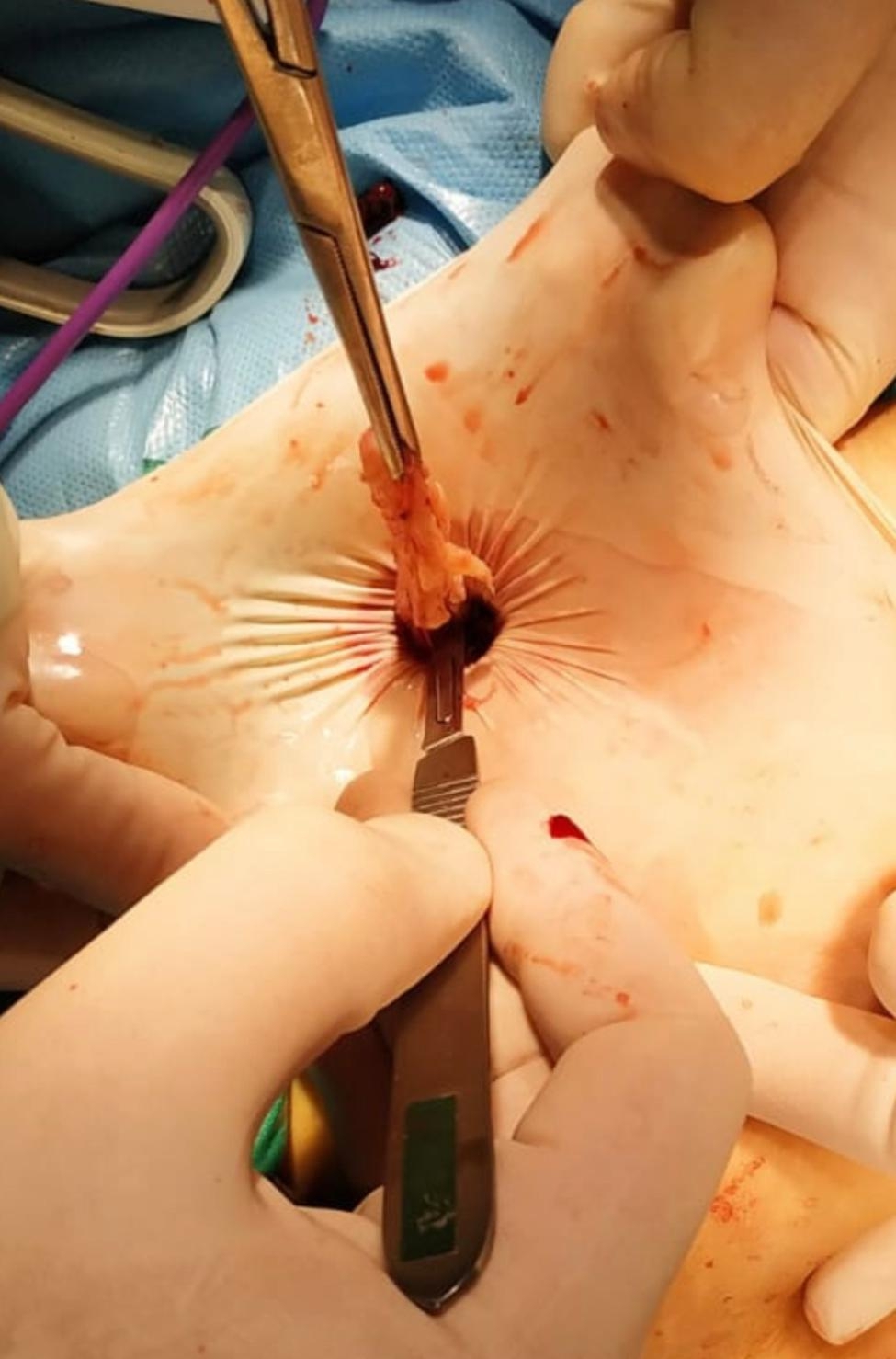
Scalpel morcellation via a 1.5 cm aperture

After exteriorization of the myoma from about a 1.5 cm aperture (Fig. 5), it was grasped with toothed pens and subjected to gradual morcellation by scalpel by cautious C-coring under visualization of an optic camera to be sure about any damage to the bag. After morcellation, all glove bags and endo-bags were inspected with visually and using methylene blue for tightness and leakage respectively.

In women undergoing PM, the left 5-mm lateral trocar incision was enlarged to 15 mm for insertion of the morcellation bag and morcellator. A medium-sized Morsafe® bag was introduced through the left incision without the trocar sheet. After the myoma was placed in the bag, the edges of the container were exteriorized via the same incision, while its caudal part was exteriorized from the umbilical trocar site by a grasper. The 10-mm umbilical sheet was reinserted into the bag. The bag was insufflated until 20 mm Hg intra-bag pressure was achieved. Versator® (VeolMedical Technologies) was then introduced from the left-sided incision, and power morcellation was performed in the container under laparoscopic visualization. After morcellation, the fascia was closed by the Easy Close® port closure system using 0 polyglactin 910 (Vicryl®).

Postoperatively, I.V. infusion of paracetamol (1000 mg) was given for postoperative pain every 8 h until discharge for all patients. Additional analgesia was given at the patient’s request as a 75 mg diclofenac I.M. injection. No narcotic analgesic was given.

All patients were ambulated between the 8 and 24 h after the operation. On postoperative day 1, the Foley catheter was removed. The timing of patient discharge was recorded as postoperative hours. Patients demonstrating spontaneous micturition without retention, the regular passage of flatus, and self-mobilization were discharged.

### Statistical analysis

The normality of the distribution of continuous variables was tested by the Shaphiro-Wilk test. The student t-test (for normal data) and Mann-Whitney U test (for non-normal data) were used to compare numerical variables between the two groups. The Chi-square test was used to investigate the relationship between categorical variables. Mean ±, standard deviations (mean ± SD), normal data, and median and interquartile for non-normal data were given as descriptive statistics. Statistical analysis was performed with SPSS for Windows Version 24.0, and a *p*-value < 0.05 was accepted as statistically significant.

## Results

A total of 61 patients (33 patients in the AMM group and 28 in the PM group) were included in the final analyses. Demographics and baseline characteristics were similar in both groups (Table 1). The largest myoma diameter was 14 and 15 cm in the AMM and PM groups respectively. There were six patients whose myoma had a diameter of 10 cm or bigger in the AMM group; there were nine corresponding patients in the PM group. The highest myoma weight was 404 and 438 g in the AMM and PM groups, respectively. No patients had undergone excessive adhesiolysis in both groups.

**Table 1:** Comparison of socio-demographic characteristics of patients

	AMM (n = 33)	PM (n = 28)	*p* value
Variables	Mean ± SD	Mean ± SD	
**Age**	28.45 ± 5.57	28.43 ± 4.77	0.985
	**Median[25-75%]**	**Median[25-75%]**	
**BMI kg/m** ^**2**^	20.7 [19.7–24.1 ]	21.55 [20.2 -24.75 ]	0.314
**Gravida**	1 [0–2 ]	1 [0–3 ]	0.481
**Parity**	1 [0–1 ]	1 [0–2 ]	0.836
**Preoperative hb**	11.5 [10.3–12.6 ]	11.9 [11.1 -12.65 ]	0.318
**Myoma mean diameter (cm)**	7 [6–9 ]	7.75 [6.45–9.75 ]	0.384
**Myoma mean weigth (gr)**	134 [99–227 ]	189.5 [117.5 -278.5 ]	0.171
	**n (%)**	**n (%)**	**p value**
**Myoma location**			
Intramural	13 (39.4)	12 (42.9)	0.937
Pedunculated	2 (6.1)	2 (7.1)	0.879
Subserosal	18 (54.5)	14 (50)	0.546
**Type of delivery**			
C/S	14 (42.4)	9 (32.1)	0.673
NSD	7 (21.2)	8 (28.6)	0.978
Nulliparous	12 (36.4)	11 (39.3)	0.897
**Prior Pelvic Surgery**			
Appendectomy	3 (9.1)	2 (7.1)	0.325
C/S	14 (42.4)	6 (21.4)	0.234
Ovarian cystectomy	3 (9.1)	4 (14.3)	0.765
Surgery	13 (39.4)	16 (57.1)	0.657

The morcellation time and total operation time were significantly lower in the AMM group compared to those in the PM group (Table 2). The mean duration time to introduce the glove bag (or endo bag) was less than 1 min, while the complete placement of PM equipment required about 11.7 min. Other surgical outcomes, which were similar between groups, included delta hemoglobin, length of hospital stay and VAS score at 12 and 24 h postoperatively. 14 patients (42.4%) in the MM group and 13 (46.4%) in the PM group requested additional analgesia. There were no per- or postoperative complications in both group with no conversion to laparotomy. One patient was transfused with two units of erythrocyte suspension postoperatively in the PM group. Sarcoma was not diagnosed in any of the cases in both groups.

**Table 2:** Comparison of surgical outcomes between groups

	AMM (n = 33)		PM (n = 28)	*p* value
Variables	Mean ± SD		Mean ± SD	
**Delta Hb**	0.9 ± 0.3		1.05 ± 0.2	0.425
**Total Operation Time (min)**	100 ± 24.3		127 ± 33.1	0.001*
**Morcellation Time (min)**	18 ± 9.2		37.4 ± 14.1	0.001*
**VAS 12 h**	4.9 ± 1.4		5.2 ± 1.7	0.678
**VAS 24 h**	2.3 ± 1.1		2.9 ± 1.6	0.455
**Length of Hospital Stay (hours)**	25 ± 1		26 ± 1	0.456

The morcellation-related mean cost per case was significantly lower in the MM group (Table 3). In six patients an Endo Catch® was used instead of a glove bag because of the myoma size (10 cm or larger). In one case, the glove bag was breached during exteriorization, and a new glove was used (it was added to the cost analysis).

**Table 3:** Comparison of costs between groups

	MM (n = 33)	PM (n = 28)	p value
Cost Variables	US Dollars	US Dollars	
**Power Morcellator per case (rent)**	-	200	
**PM bag per piece**	-	150	
**8-sized latex-free gloves per piece **	0.5	-	
**Endo bag per piece (6 patients)**	30	-	
**Total morcellation cost for one case**	5.9	350	0.001*

## Discussion

After FDA warning statement, we preferred to routinely utilize contained PM morcellation or in-bag AMM in laparoscopic myomectomy. The current study mainly revealed that total operation and morcellation time were significantly shorter for patients who had undergone laparoscopic myomectomy with in-bag abdominal manual morcellation compared to contained power morcellation, with costs substantially affordable. However, the other surgical outcomes (Delta Hb levels, length of hospitalization, VAS intensity at 12 and 24 h postoperative times, need for additional analgesic and complications) were determined as similar.

There is a paucity of studies comparing in-bag AMM and PM in the literature. Frascà et al. compared uncontained PM morcellation with in-bag AMM during laparoscopic myomectomy for myomas between 4 and 10 cm in mean diameter and reported that in-bag AMM was related to statistically significant longer surgical times compared to uncontained PM for both morcellation time (9.47 min versus 6.16 min) and total operation time (113.24 min versus 96.74 min) in a randomized controlled trial [14]. They morcellated the myoma by either scalpel or scissors, which had been placed into an Endo bag pulled out through a 20 mm lower central incision that had been previously enlarged. Ventruella et al. reported that in-bag AMM (via a 30-mm central incision) was related to longer mean morcellation (16.18 min versus 14.35 min) and total operation times (96.96 min versus 92.07 min) without being statistically significant, compared to uncontained PM, as well [15].

Although we performed nearly similar AMM techniques to those described in these studies, our use of a left trocar incision and a glove bag for the extraction of myomas smaller than 10 cm was a point of difference, as previously, a central trocar incision had been used. Our mean morcellation time and total operation time in the AMM group were also longer compared to their results. In our opinion, these differences were related to the mean myoma size differences between the studies. In this study, the mean size of the myomas was larger than the mean myoma sizes, which were reported in these two studies. More importantly, we did not exclude myomas larger than 10 cm which they had done. There were six myomas larger than 10 cm in our AMM group. Besides, it is our opinion that the use of either a glove bag or an Endo bag has the same effect on surgical times because in the case of both methods, the time required for placing the bag in the abdominal cavity, putting the myoma into the bag and exteriorizing the bag are similar.

Another comparative study (comparing AMM versus PM) used a larger incision of 4 cm for manual myoma morcellation in a robotic myomectomy, reported that contained AMM was related to a statistically significant shorter operative time (105.39 min versus 126.11 min) compared with uncontained PM [16]. According to the authors, increased incisional length shortens surgical times dramatically; although the operating time, which was reported by Sanderson, was similar to our result, we used smaller incisions of about 2 cm, which provides an aperture of about 1.5 cm for morcellation during AMM. Hence, it is our opinion that enlarging the incision is important but not mandatory for reducing surgical times during AMM.

Regarding in-bag AMM safety, in the above-mentioned three studies, authors reported that AMM and PM were associated with similar surgical outcomes such as blood loss, postoperative pain, and hospital stay without causing any significant complications. Our study supported their findings.

Surgical gloves have, over the years, been described as safe and effective retrieval bags instead of costly specially designed equipment in laparoscopic surgery [17, 18]. Glove bags are widely used in gynecological laparoscopic surgery as well because the ability to manipulate a glove bag inside the abdomen is acquired during residency, with most gynecologists familiar with this approach [19, 20]. Hence, we think the use of a glove bag is not an innovative technique for manual myoma retrieval, but it is often ignored.

Akdemir et al. described a contained PM technique, which used a latex glove as a container in myomas with a maximum diameter of 10 cm [21]. They concluded that a surgical glove is strong enough to withstand the high gas pressure and manipulation without easily perforating during enclosed PM. They also reported that the elastic texture of the glove allows it to expand and be used with different myoma sizes. In our study, with myomas smaller than 10 cm, we could easily place them into the glove bag and morcellate them. Only in 1 case did the glove bag tear during the exteriorization of the myoma and we used a new one. In our opinion, a glove bag is an efficient substitute for an Endo bag in that it retains its integrity during scalpel or scissors morcellation. In an in-vivo study, different morcellation techniques (manual morcellation, single-site power morcellation, and double-site morcellation) were compared. According to the results, although in-bag manual morcellation requires longer incisions, it has been reported to be sufficiently safe, especially in terms of bag integrity [22].

## Conclusions

In conclusion, the in-bag AMM (both glove bag or Endo bag) or contained PM for specimen extraction in laparoscopic myomectomy resulted in similar outcomes in terms of delta hemoglobin, postoperative pain intensity (VAS score at 12 and 24 h postoperatively), the need for additional analgesia and the length of hospital stay; however, total operation time and morcellation time were significantly shorter in the AMM group, indicating a prominent advantage. Significant cost-effectiveness is also a critical advantage of in-bag AMM compared to containing PM. Both the techniques: in-bag AMM and contained PM were found to be safe, as demonstrated by the lack of significant complications in relation to the respective methods employed. Further randomized large-scale trials comparing glove in-bag AMM and contained PM in laparoscopic myomectomy are required.

## Data Availability

All data is contained within the manuscript. The datasets used and analyzed during the current study available from the corresponding author on reasonable request.

## List of Abbreviations

AMM: Abdominal manual morcellation
BMI: Body mass index
PM: Power morcellation
VAS: Visual analogue scale.
